# Gene Therapy with Chitosan Nanoparticles: Modern Formulation Strategies for Enhancing Cancer Cell Transfection

**DOI:** 10.3390/pharmaceutics16070868

**Published:** 2024-06-27

**Authors:** Varvara Antoniou, Elena A. Mourelatou, Eleftheria Galatou, Konstantinos Avgoustakis, Sophia Hatziantoniou

**Affiliations:** 1 Pharmacy Program, Department of Health Sciences, School of Life and Health Sciences, University of Nicosia, Nicosia 2417, Cyprus; antoniou.v3@live.unic.ac.cy (V.A.); galatou.e@unic.ac.cy (E.G.); 2 Bioactive Molecules Research Center, School of Life and Health Sciences, University of Nicosia, Nicosia 2417, Cyprus; 3 Laboratory of Pharmaceutical Technology, Department of Pharmacy, School of Health Sciences, University of Patras, 26 504 Rion, Greece; avgoust@upatras.gr (K.A.); sohatzi@upatras.gr (S.H.)

**Keywords:** chitosan, chitosan nanoparticles, gene therapy, cancer gene therapy, cancer cell transfection

## Abstract

Gene therapy involves the introduction of exogenous genetic material into host tissues to modify gene expression or cellular properties for therapeutic purposes. Initially developed to address genetic disorders, gene therapy has expanded to encompass a wide range of conditions, notably cancer. Effective delivery of nucleic acids into target cells relies on carriers, with non-viral systems gaining prominence due to their enhanced safety profile compared to viral vectors. Chitosan, a biopolymer, is frequently utilized to fabricate nanoparticles for various biomedical applications, particularly nucleic acid delivery, with recent emphasis on targeting cancer cells. Chitosan’s positively charged amino groups enable the formation of stable nanocomplexes with nucleic acids and facilitate interaction with cell membranes, thereby promoting cellular uptake. Despite these advantages, chitosan-based nanoparticles face challenges such as poor solubility at physiological pH, non-specificity for cancer cells, and inefficient endosomal escape, limiting their transfection efficiency. To address these limitations, researchers have focused on enhancing the functionality of chitosan nanoparticles. Strategies include improving stability, enhancing targeting specificity, increasing cellular uptake efficiency, and promoting endosomal escape. This review critically evaluates recent formulation approaches within these categories, aiming to provide insights into advancing chitosan-based gene delivery systems for improved efficacy, particularly in cancer therapy.

## 1. Introduction

The ability to intervene in the human genome and modify specific regions therein has been a primary goal of medicine since the recognition of DNA as the fundamental unit of heredity [1]. The initial conceptualization of replacing or correcting a single gene responsible for a disease with the aim of therapeutic gene enhancement, commonly referred to as gene therapy, was first noted in 1970 [2]. One of the earliest attempts to implement this concept occurred in 1990, when a viral vector was employed to deliver the gene encoding adenosine deaminase (ADA) to the T cells of a four-year-old girl with severe combined immunodeficiency (SCID). The transduction of cells was performed ex vivo and the modified cells were infused back into the patients. Although this therapy did not cure the disease, it marked a milestone by reducing the severity of the disease symptoms and demonstrated the potential safety of gene therapy [3]. Three decades later, and after overcoming various safety issues, gene therapy today is considered as a therapeutic option for an increasing number of diseases. This approach is now proven to be reliable, since different and safer methods have been developed for correcting or modifying the functioning of human genes. Innovative techniques are being applied to develop therapies offering treatment possibilities for various diseases and conditions. Such diseases caused by inherited genetic disorders include cystic fibrosis, hemophilia, muscular dystrophy, sickle cell anemia, acquired genetic diseases such as cancer, and certain viral infections like acquired immunodeficiency syndrome. Several gene therapies have been approved in the United States and the European Union in the last six years, and many others targeting a variety of diseases are heading towards clinical trials [4].

Existing techniques for administering gene therapy are based on two fundamental approaches: ex vivo and in vivo therapy. Ex vivo therapy is the more common approach, where target cells (e.g., hematopoietic stem cells, T cells) from blood or other tissues are collected from the patient, genetically altered in the laboratory, and subsequently reinfused into the patient’s body. This way, potential side effects associated with the carrier used to deliver the genetic material to the target cells are avoided; hence, the safety profile of the treatment is increased. Moreover, since the cells to be modified are selected, the treatment is characterized by increased specificity. In vivo gene therapy, on the other hand, consists of a delivery system containing the desired genetic material that is administered either intravenously or topically (e.g., ocular administration) to the patient in order to transfect target cells inside the body. In this approach, there is no need to apply complex procedures to remove specific cells from the patient, thus making the process simpler, less invasive, and often with a lower cost [5]. In terms of gene manipulation, gene therapy was first performed in order to replace or repair genes that were defective. Today, many different methods are available, including gene silencing, editing, replacement, or supplementation [6]. When gene therapy is applied for cancer treatment, the goal usually is to minimize or shrink the size of a tumor by either overexpressing or silencing the targeted gene [7].

A gene therapy drug typically consists of an expression cassette generated from complementary DNA (cDNA) and carries a promoter on the 5′ end and a transcription termination and polyadenylation signal on the 3′ end. Alternatively, a gene therapy drug may also consist of an RNA sequence, depending on the gene therapy mechanism. Conventional nucleic acids lead to transcription, resulting in mRNA production for translation into a therapeutically active protein or non-coding RNAs (ncRNAs). These ncRNAs can be either micro-RNAs (miRNAs), capable of controlling gene transcription–translation processes, or small interfering RNAs (siRNA) that target specific mRNA molecules for destruction. Both miRNAs and siRNAs are capable of gene silencing for therapeutic purposes, leading to selective reduction of disease-associated proteins. However, siRNA needs to be present in the cytoplasm of cells to act effectively, and the efficient and safe delivery of these oligonucleotides into cells remains a challenge [1]. The use of the CRISPR/Cas9 molecular tool is considered a groundbreaking technique. Clustered regularly interspaced short palindromic repeats (CRISPR) and associated endonuclease Cas (Cas9) constitute a cutting-edge technology in genome editing targeting specific DNA sequences using small RNA molecules, assisting Cas9 endonuclease in gene correction responsible for genetic diseases [8].

Various carriers have been employed for the effective delivery of the genetic material into target cells, since the administration of naked DNA faces challenges such as low transfection efficiency due to poor cell internalization, attributed to its large size and hydrophilicity as well as rapid degradation by nucleases [7]. A gene carrier generally should have low toxicity, be able to effectively incorporate and protect the genetic material from enzymatic degradation, be able to avoid rapid clearance from systemic blood circulation, and target specific cells with increased cellular uptake and ability for endosomal escape [9]. Carriers can be classified into two groups: viral and non-viral vectors. Viral vectors (such as adenoviruses (AdV), lentiviruses (LV), adeno-associated viruses (AAV), retroviruses (RV), and herpes simplex virus) are the vectors most commonly used in gene delivery (especially AAV) and in most clinical trials for gene therapy [10]. This is attributed to the high transfection efficiency achieved with the use of viral vectors. Nevertheless, a number of disadvantages, such as inflammation or cytotoxicity caused by immune responses triggered by the immunogenicity of viral vectors; insertional mutagenesis, i.e., the random insertion of viral genetic material into the human genome, resulting in an undesired mutation; as well as more complex and costly production process [11], have led researchers to seek alternative gene carriers.

Non-viral vectors, although less effective in transfection, demonstrate lower toxicity, have the ability to effectively carry various genetic materials without size restrictions and with high load, can be produced in large scale, and can be structurally modified to target specific cells [12]. There are two main categories of non-viral vectors for gene delivery based on their structural materials, lipidic and polymeric. Additionally, peptides, inorganic materials, and hybrid systems can also be employed. Most commonly, positively charged molecules (lipids or polymers) formulated as nanoparticles (NPs) are used to form electrostatic bonds with the negatively charged genetic material, thus creating compact complexes, namely, lipoplexes and polyplexes, respectively [13]. Due to their small size, NPs are suitable for crossing cellular membranes and are capable of enhancing the pharmacokinetics, pharmacodynamics, and biodistribution of therapeutic molecules [14].

The mechanism of gene transfer from polyplexes to eukaryotic cells involves specific stages and depends largely on the size and properties of each polymer. These stages include the condensation of polymer with DNA via electrostatic interactions (DNA condensation), cellular uptake through endocytosis, release from endosomes [15], detachment of DNA from the carrier, and DNA transfer to the nucleus, where gene transcription occurs. Understanding these processes is paramount for optimizing gene transfer [16]. NPs enter mammalian cells via different types of endocytosis, such as micropinocytosis and phagocytosis into endocytic vesicles or endosomes, or via fusion [10].

Various types of polymers have been used as gene delivery systems and can be classified into natural polymers, which include chitosan (Ch) and atelocollagen, and synthetic polymers, which include poly-L-lysine (PLL), polyethyleneimine (PEI), and dendrimers. Synthetic polymers exhibit high transfection efficiency, but the primary concern associated with their use is in vivo toxicity due to their non-targeted action and cellular accumulation after repeated administration. Thus, their clinical use is limited, and consequently, the use of biodegradable polymers for gene transfer, such as Ch, has gained ground in recent years, offering significant advantages. Moreover, they can be easily modified by introducing targeting molecules to target specific cells and demonstrate high in vivo effectiveness in cell transfection. Their drawbacks include relatively slow biodegradation and rapid removal from the bloodstream after parenteral administration [17].

Most of the gene therapies approved by the FDA and EMA to this day are using viral vectors, especially AAV (e.g., Glybera for familial lipoprotein lipase deficiency, Luxturna for retinal dystrophy, Zolgensma for spinal muscular atrophy) and RV (e.g., Zalmoxis for restoring immune system after HSCT, Strimvelis for severe combined immunodeficiency, Yescarta for large B-cell lymphoma). Out of the non-viral carriers, lipidic nanoparticles have gained much attention, leading to the first approved product by the FDA and EMA in 2018, named Onpattro, for the treatment of hereditary transthyretin amyloidosis (hATTR) by using siRNA, and the recent vaccines for COVID-19, where lipid nanoparticles were used as mRNA carriers [18,19]. The reason for seeing more viral than non-viral marketed products is their superior transfection efficiency and cell uptake, although lipid nanoparticles are more advantageous in terms of safety profile, loading efficiency, and size of gene material that they are able to carry [20].

## 2. Chitosan in Gene Delivery

### 2.1. Chitosan Structure and Properties

**Figure 1:**
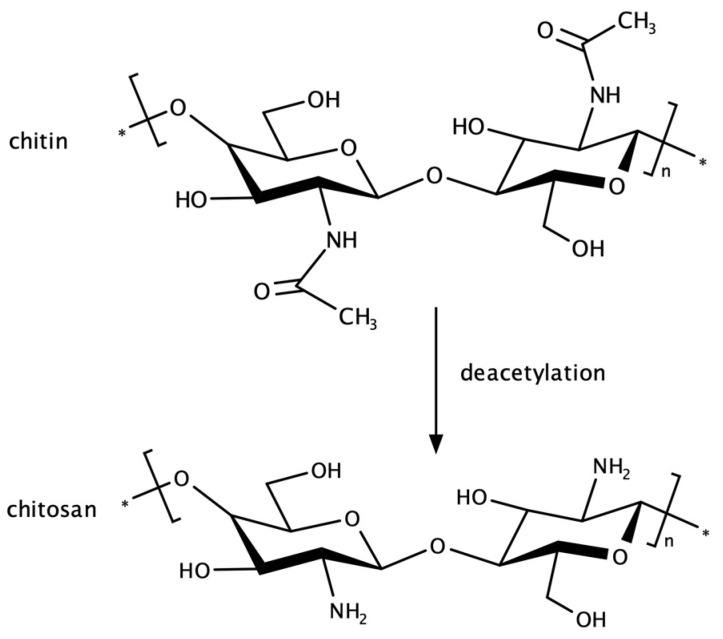
Chitosan production from chitin. * indicate the points at which the polymer chain continues, denoting the repetition of the enclosed unit.

Chitosan (Ch) is a natural polymer, also known as poly(2-deoxy-2-amino-D-glucopyranose). It consists of residues of 2-deoxy-2-amino-D-glucopyranose connected by β1→4 bonds, creating repeating units of D-glucosamine and N-acetyl-D-glucosamine linked by glycosidic bonds. It is obtained through enzymatic or chemical deacetylation of chitin, a natural polysaccharide abundantly found in nature (Figure 1). Chitin serves as the primary structural material in the exoskeletons of arthropods, such as crabs and shrimps, as well as in the cell walls of fungi [21].

Ch is used in various biomedical applications such as tissue engineering and drug and gene delivery. It is one of the most widely studied biopolymers in drug and gene therapy due to its significant advantages such as biocompatibility, absence of cytotoxicity, biodegradability, and mucoadhesive and antimicrobial properties. Moreover, chitosan nanoparticles (ChNPs) exhibit good stability in serum and prolonged circulation time in the body [22]. The degradation products from the in vivo enzymatic degradation of Ch are non-harmful since they are endogenous substances, like water and carbon dioxide. Additionally, ChNPs are effective as carriers in gene delivery while being economically and environmentally friendly. Chitin, from which Ch is derived, is an abundant by-product of the food processing industry; thus, large quantities of this polymer are available for biomedical use at minimal expense [23].

The D-glucosamine units possess primary amino groups that, under a given pKa (6.5), become protonated, thereby imparting multiple properties of ChNPs for gene delivery. These properties include the ability to form complexes with negatively charged nucleic acids, increased mucoadhesion where the mucin barrier needs to be overcome, enhanced cellular and nuclear uptake after endosomal escape, and the ability for chemical modifications on D-glucosamines to enhance the chemical properties of Ch. Cellular and nuclear membranes are negatively charged, and thus, interaction with positively charged Ch leads to the uptake and transport of the cargo into the nucleus [8]. Additionally, cationic polymers like Ch, due to their ability to “capture” protons from their amino groups, have the capability of endosomal escape known as the “proton sponge” phenomenon: nucleic acid–polymer complexes enter cells and become entrapped within the endosomal membrane. As the pH of the endosome changes, the cationic polymer becomes protonated, leading to the influx of chloride ions and water into the endosome. Eventually, osmotic pressure becomes strong enough to rupture the endosomal membrane, allowing nucleic acid to escape into the cytoplasm [24].

### 2.2. Chitosan in Cancer Treatment

ChNPs have been widely studied for drug and gene delivery in cancer treatment. Cancer represents a major public health issue globally, accounting for more than one-tenth of all deaths worldwide. Conventional cancer treatment approaches include surgery, radiotherapy, and chemotherapy, with chemotherapy remaining one of the most significant therapies for most cancer patients. However, chemotherapy faces numerous drawbacks related to multiple adverse effects, which limit its therapeutic efficacy, decrease the quality of life, and worsen cancer prognosis. In order to mitigate these side effects and improve treatment efficacy and patient compliance, better-targeted therapies are required [22]. Cell targeting with ChNPs can be achieved in various ways, such as passive and active targeting. Passive tumor targeting is obtained via the phenomenon of the Enhanced Permeability and Retention effect (EPR), where NPs exploit the unique pathophysiological characteristics of tumors as they extravasate into the tumor tissue via the leaky vasculature and subsequently accumulate in the tumor site due to reduced lymphatic drainage [25]. On the other hand, active targeting can be obtained through receptor-mediated targeting or responsiveness to stimuli. Targeting a receptor overexpressed in tumor cells, such as folate, can be achieved by attaching targeting molecules, e.g., folate, to the surface of ChNPs for ligand-receptor targeting. Stimuli-responsive refers to the strategic design of ChNPs in such a way that they respond to specific stimuli, both external and internal, characteristic of the tumor microenvironment. This allows for controlled release of genes/drugs only in targeted cancer cells, while minimizing effects on healthy cells. An example of an internal stimulus is pH. The pH value of normal tissues and blood is approximately 7.4, while pH values in tumor tissues are slightly acidic (6.5–7.2). Lysosomes are more acidic, with a pH of 4.5–5. Therefore, ChNPs can be modified to respond to the acidic pH of the tumor microenvironment, leading to targeted drug or gene release [26]. An example of an external stimulus is ultrasound [27]. Furthermore, Ch may accumulate in the tumor area, initiate polarization of M1 macrophages, and convert the immunosuppressive tumor microenvironment to immunosupportive, thereby exerting anticancer action and enhancing the effectiveness of cancer immunotherapy. Moreover, Ch can activate innate immune responses to exert its anticancer action, while it can also inhibit the growth of cancer cells, angiogenesis induced by tumors, and tumor metastasis. Therefore, Ch and its derivatives can be useful in cancer therapy through multiple pathways [28].

### 2.3. Factors Affecting Loading Efficiency of Gene Cargo to ChNPs and Their In Vivo Performance

Crucial factors affecting the in vitro and in vivo applications of ChNPs are their physicochemical properties, such as size, surface charge, shape, and surface morphology, that are closely related to Ch properties. Specifically, molecular weight (MW), degree of deacetylation (DD), chemical modifications that alter Ch properties, and the ratio between positively charged nitrogen in the amine groups in Ch to the ratio of negatively charged phosphate groups in the genetic material (N/P ratio), play pivotal roles in Ch performance [20]. MW can affect the solubility of Ch [29], the size, stability and cell internalization of ChNPs, the subsequent release of their genetic cargo in the cytosol, and the transfection efficiency achieved with ChNPs [30,31]. When the MW of Ch is low, then the complexes formed with nucleic acids are not stable. To obtain stable complexes, where the nucleic acids are also protected against enzymatic degradation, Ch’s MW should be high enough, depending also on the genetic material to be complexed (e.g., 65–170 kDa for siRNA). Additionally, an increase of Ch’s MW can lead to increased NP size, which in turn affects cell uptake [20]. When the size of NPs is significantly increased, then the cellular uptake decreases, causing a reduction in transfection efficiency [32]. The selection of MW should be performed with caution since, although high MW leads to the formation of complexes with increased stability, their size is also increased and the release of the genetic material in the cytosol is constrained due to strong binding, thus slowing down transfection efficiency [20]. Lowering MW can be compensated for with a simultaneous increase in the DD to obtain optimum transfection efficiency [32].

The DD affects the number of amine groups and hence the overall positive charge of Ch at a specific pH, which in turn affects its solubility, its ability to form a complex with the genetic material, cell internalization, and endosomal release [20,33]. For gene delivery applications, it is preferred to use Ch with a high DD (higher than 65% for pDNA, higher than 80% for siRNA) which leads to increased binding efficacy of nucleic acids, as well as higher cellular uptake. Moreover, if the DD changes, at a specific MW, then the N/P ratio that is needed to obtain full complexation of nucleic acids also changes, with a higher DD requiring a lower N/P ratio [20,32].

N/P ratio is a parameter determining the final overall charge of the complex formed between Ch and the genetic material, with high values indicating NPs with positive charge and low values neutral to negatively charged NPs. As a result, the N/P ratio will affect the stability of the complexes, as increased charge leads to higher stability due to electrostatic repulsions, and the interactions with cells and transfection efficiency [20]. The N/P ratio that is needed to obtain full complexation of nucleic acid depends on the MW and DD of Ch, as stated above. If the MW is low, then a higher N/P ratio is required to obtain a stable complex with nucleic acids, compared to high-MW Ch of the same DD. Moreover, a positive charge can also improve movement in the cytoplasm towards the nuclear membrane and high N/P ratios, indicating higher Ch concentration, and can lead to improved endosomal release, due to an increase of osmotic pressure inside endosomes [32]. On the other hand, a high N/P ratio can hinder the release of nucleic acid in the cytosol, due to strong condensation, indicating the need for achieving a balanced formulation by carefully selecting the individual parameters [20].

Other parameters that affect the loading efficiency of nucleic acids in ChNPs are the pH and ionic strength of the surrounding medium, as well as the method used for the preparation of the NPs. pH affects the complexes formed, since it affects the protonation of the Ch’s amino groups, with complex formation being favored under acidic conditions. An increase in pH leads to a reduction in Ch’s charge, causing problems with Ch solubility as well as nucleic acid dissociation from complexes, due to the weakened electrostatic interactions (although hydrophobic interactions and hydrogen bonds can maintain a certain degree of association under such conditions) [20]. Such phenomena should be taken into consideration, not only for the preparation of the NPs, but also in respect to their in vivo performance, since changes in the pH could lead to complex destabilization and reduced transfection efficiency.

Various preparation methods have been employed for the formation of nucleic acid–Ch complexes, such as simple complexation or ionic gelation (entrapment or surface absorption). Simple complexation is performed by the addition of Ch solution to an equal volume of nucleic acid solution, where the concentrations of the solutions have been adjusted to achieve a specific N/P ratio through mixing and incubation at room temperature. Ionic gelation involves the usage of a polyanion as a crosslinker (usually sodium tripolyphosphate (TPP)) for the formation of NPs through electrostatic interactions. In this case, nucleic acids can be either entrapped in the NPs by adding nucleic acids in the solution of TPP before NPs are formed, or absorbed on the surface of ChNPs by adding nucleic acid after the NPs are formed. The chosen method can affect the transfection efficiency achieved through changes in the stability and size of the produced complexes, with entrapment of nucleic acid via ionic gelation leading to more stable complexes [34].

Improvement of Ch’s properties can be performed via chemical modifications of Ch’s functional groups such as primary amines as well as primary and secondary hydroxyl groups. Most commonly, this approach aims to improve Ch’s solubility at neutral pH with the addition of trimethylated groups, carboxymethyl groups, or polyethylene glycol (PEG). Moreover, structural modification can be employed to improve cancer cell targeting by adding targeting molecules to Ch [23]. These are discussed in more detail in the following sections.

### 2.4. ChNP Cellular Uptake, Intracellular Trafficking, and In Vivo Distribution

The complex design of the cell membrane acts as a strong barrier to most substances, hindering the activity of various therapeutic molecules that are unable to cross this barrier. Phospholipids with negatively charged head groups are responsible for the net negative charge in the plasma membrane of mammalian cells, and because of this, cationic polysaccharides like Ch may bind to cell membranes with ease through electrostatic interactions, increasing cellular absorption. There are various pathways through which ChNPs enter cells, depending on their size, surface charge, surface modification, hydrophobicity, and cell type. Cell internalization of ChNPs takes place predominantly through endocytosis, due to their charge. Depending on their size, endocytosis can take place either as phagocytosis (for NPs > 250 nm) or as pinocytosis, with the latter being affected not only by size but also by surface characteristics such as charge and the presence of ligands [35].

Pinocytosis uptake can be performed through micropinocytosis or receptor-mediated endocytosis, which is the most common pathway for cellular uptake. In micropinocytosis, NPs are internalized in micropinosomes, which acidify after entering the cell and can later on fuse with late endosomes or lysosomes, or recycle onto the cell membrane. In receptor-mediated endocytosis, where the internalization process is triggered by the binding of targeting ligands attached on the surface of NPs to receptors overexpressed on target cells, two main mechanisms related to NP internalization can be identified: clathrin-mediated endocytosis or caveolin-mediated endocytosis. In clathrin-mediated uptake, the initial clathrin-coated vesicles that are formed from the plasma membrane lose their clathrin coat, fuse with early endosomes, then turn into late endosomes that finally fuse with lysosomes. In caveolin-mediated uptake, caveolin vesicles, created by plasma membrane domains rich in cholesterol and caveolin, fuse to create caveosomes that can later fuse with early endosomes. In this pathway, degradation is avoided since caveosomes’ cargo doesn’t end up in lysosomes. From caveosomes, the cargo can be transferred to the Golgi apparatus, endoplasmic reticulum, or cytoplasm. The main pathways through which ChNPs are uptaken by cells are clathrin-mediated endocytosis and micropinocytosis, with caveolin-mediated endocytosis playing a secondary role [36]. Surface modification, such as attachment of hydrophobic chains to ChNPs, can increase the cellular uptake by cancer cells. Moreover, NPs that are larger in terms of size are endocytosed through pathways that are energy dependent, in contrast to smaller sized NPs that exhibit a more passive uptake that is independent of energy [35].

After cell uptake, ChNPs should be able to escape from endosomes or lysosomes into the cytoplasm, in order to deliver their genetic cargo to the target organelle. In some cases, ChNPs release the genetic material in the cytoplasm, whilst in other cases, ChNPs can enter the nucleus. Endosomal/lysosomal escape is crucial in this process, as well as avoiding degradation in lysosomes due to enzymes and low pH. Endosomal escape is performed through the “proton sponge” mechanism. Initially, nucleic acid–cationic polymer complexes enter cells and become trapped within the endosomal membrane. As the endosomal pH changes, the cationic polymer becomes protonated, leading to an influx of water and chloride ions into the endosome. Eventually, osmotic pressure becomes strong enough to rupture the endosomal membrane, allowing the escape into the cytoplasm [36]. Lysosomal escape takes place via the proton sponge effect as well, in a manner similar to the one described previously [35].

ChNPs carrying DNA need to deliver it to the cell nucleus. Depending on their size, ChNPs can enter the nucleus with either passive or active transportation through the nuclear envelope. Small NPs enter in a passive manner, whilst larger NPs enter through the nuclear localization mechanism, where short peptide sequences called nuclear localization signals (NLS) bind to nuclear transport receptors which transport the NPs into the nucleus. For this purpose, ChNPs need to be functionalized with NLS peptides. Nevertheless, the effectiveness of transportation can be different in cancer cells, since in these cells, the nuclear import route is damaged [37]. Moreover, ChNPs can enter the nucleus at the end of mitosis after the nuclear envelope is reassembled [38].

In vivo, ChNPs demonstrate the ability to protect nucleic acids and prevent degradation from nucleases, with the DD and MW of Ch playing a significant role. In order to obtain tumor targeting, either by passive or active targeting, prolonged circulation time needs to be established. Circulation time can be affected by the physicochemical characteristics of ChNPs, such as size, charge, MW, and DD, and can be prolonged through surface modification (such as conjugation with PEG, which reduces recognition and uptake by the reticuloendothelial system (RES). Due to RES activity, ChNPs tend to accumulate in the liver and spleen, but, with appropriate surface modification, tumor tissues can be targeted to maximize therapeutic effectiveness and minimize systemic toxicity. Surface modification can also aid in reducing opsonization, i.e., the binding of opsonins on the NPs’ surface, which aims at marking the NPs for clearance by the immune system [38]. Further discussion on the types and functionalities of surface modifications and/or Ch chemical modifications is provided in the following sections.

### 2.5. Clinical Applications of Chitosan

Clinically, Ch is used as an excipient in wound dressing since it is generally recognized as safe (GRAS), and is used in several approved hemostatic products, such as Axiostat^®^, Celox^TM^, ChitoFlex and Anscare ChitoClot, and products for wound care, such as SynePure^TM^ and Catasyn^TM^. Numerous clinical trials have been conducted, or are still ongoing, for the application of Ch in treating oral or dental infections, osteoarthritis, diabetic foot ulcers, and wound care; in ocular drug delivery; in textiles for treating skin conditions; in reducing fat absorption; as immunoadjuvant in cancer therapy; as a hemostatic; as a barrier to prevent postsurgical adhesion; and in dietary supplements with uses in various conditions such as obesity, dyslipidemia, gastro intestinal tract disorders, and others [38].

However, no clinical trial has yet been conducted on ChNPs as gene carriers. Most of the research performed in this area concerns preclinical studies, with more than half being conducted solely in vitro. This is a major drawback, since the gene therapy results obtained through in vitro studies are often not validated in in vivo studies, mainly due to genetic variations in living organisms that are not easy to predict but directly affect the effectiveness of gene therapeutics. Despite the numerous advantages that ChNPs have for gene delivery, the lack of clinical trials is attributed to the poor transfection efficiency they exhibit in vivo, which is influenced by various factors such as problematic solubility, stability, targetability, cell uptake, and intracellular escape. These areas are attracting the interest of researchers to address these issues and provide formulations of ChNPs with increased in vivo transfection efficiency. Moreover, since the raw material used for Ch production, i.e., chitin, is derived from shellfish, impurities such as proteins, bacteria and bacterial endotoxins, heavy metals, and other contaminants can cause safety concerns, making the purity of the raw material of outmost importance for clinical applications. Additionally, different Ch sources and manufacturing processes can introduce variability in the properties of the final product. Hence, to guarantee safety and consistency, Ch’s production and purification processes must be closely examine,d and guidelines for the manufacturing of Ch of appropriate grade should be established [38,39]. Finally, genetic drugs are still in their early stages of development, with three products being approved so far by the FDA and EMA for gene therapy for cancer treatment, concerning mainly melanoma and lymphoma [18].

Overall, Ch-based gene therapy medications have a bright future ahead of them, even though there is still a long way to go before they are actually exploited. Ch is a promising alternative to lipid nanoparticles owing to its high loading capacity; excellent biocompatibility and low toxicity; modifiable nature which allows improvement of its properties, such as enhancing its solubility, stability, and targeting capabilities; as well as its ability to overcome cellular barriers, thus facilitating the intracellular delivery of nucleic acids. Although lipid nanoparticles are the most popular non-viral vector today (apart from naked DNA, which exhibits fast degradation and low transfection efficiency and cell targeting), they are still facing challenges such as possible dose-dependent toxicity, attributed to the positive charge of phospholipid headgroup, and the need for repeated administration due to lack of long-term knockout effects [20]. The targeting and efficacy of Ch have been shown in several in vivo animal models, highlighting Ch’s ability to achieve targeted delivery and sustained gene expression. Understanding the mechanisms and means to overcome cellular barriers in the transport of nucleic acids with ChNPs, as well as designing strategies for enhanced in vivo cellular transfection, will further promote the development of gene delivery using them, and therefore the full utilization of the advantages offered by these carriers compared to other gene carriers, thus unlocking their potential to aid in clinical gene therapy.

In this review, the most recent advancements for improving cancer cell transfection by ChNPs within the context of cancer gene therapy are analyzed and evaluated through a comprehensive overview of existing literature, enabling readers to quickly get up to date with the latest advancements, methodologies, and challenges in the field. NPs where Ch was used merely for coating and articles solely concerning drug delivery via NPs without nucleic acids were not included in this study. The different approaches are grouped and categorized for highlighting emerging trends, while at the same time, gaps in current research are identified; thus, researchers can identify and focus their efforts on the most promising and underexplored areas. A limitation of the current study is the dependance on existing literature where a great number of research studies have tested the formulation only in vitro and hence, the in vivo transfection efficiency of the proposed strategy cannot be verified.

## 3. Strategies for Enhanced Transfection Efficiency of Chitosan Nanoparticles (ChNPs)

**Figure 2:**
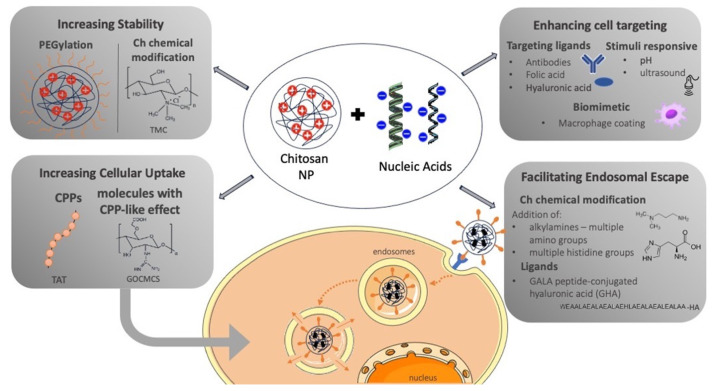
Modern strategies for improving transfection efficiency of ChNPs in cancer cells.

The most recent strategies implemented for the enhancement of transfection efficiency obtained by ChNPs in cancer cells can be categorized into five main categories: increasing ChNPs’ stability, increasing cellular uptake, enhancing tumor cell targeting, improving ChNPs’ endosomal escape, and synergistic approaches in which two or more of the previously mentioned strategies are combined (Figure 2). Characteristic studies that can be classified in one of these strategies and have been performed in the past five years are summarized in the following subsections.

### 3.1. Strategies for Increasing Stability of ChNPs

#### 3.1.1. Chemical Modification of Ch

**Figure 3:**
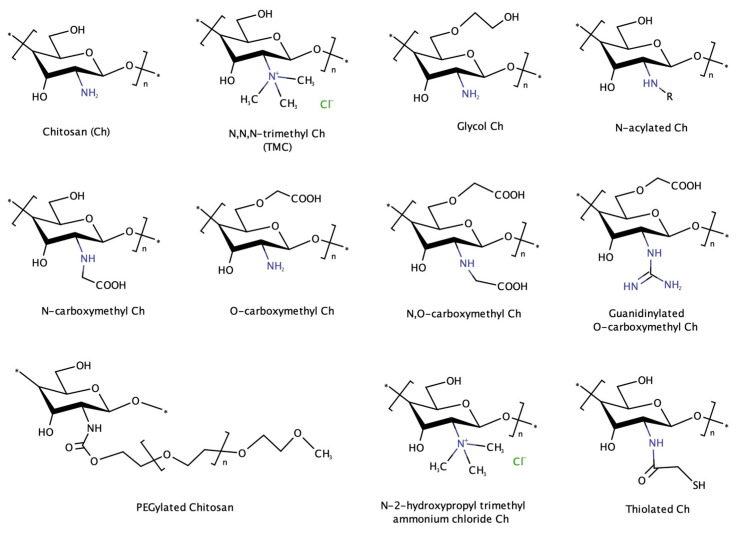
Chemical structure of Ch and Ch derivatives. * indicate the points at which the polymer chain continues, denoting the repetition of the enclosed unit.

ChNPs encounter several challenges when administered in vivo, such as unfavorable pH conditions for Ch’s solubility in the bloodstream, interactions with proteins, and enzymatic degradation. The surface charge of ChNPs is highly dependent on pH, with the amino groups of Ch being protonated at acidic pH (<6.5), leading to positive surface charge and Ch solubilization. However, in physiological pH, ChNPs may lose their positive charge, resulting in reduced stability and compromised gene delivery efficacy, since they lose their integrity and the genetic material gets released into the bloodstream [40,41]. Additionally, the interactions of ChNPs with cell membranes, which are negatively charged, is reduced; thus, their internalization into cells is hindered. To address these issues, various derivatives of Ch with cationic properties have been obtained through chemical modification of its functional groups (i.e., primary amines, and primary and secondary hydroxyl groups), such as N-acylated Ch derivatives, carboxylated Ch derivatives, and quaternary ammonium Ch derivatives, e.g., N,N,N-trimethyl Ch (TMC) (Figure 3) [42].

TMC, one of the most commonly employed Ch derivatives, has several advantages compared to Ch. Its main advantage is the ability to remain soluble across a wide range of pH, even under neutral and basic conditions, unlike Ch, while its higher positive charge, which interacts favorably with the negative charge of the cell membrane, enhances cellular uptake [43]. These attributes have been found to positively affect the transfection efficiency of polyplexes produced with TMC [44].

To further improve the stability and transfection efficiency of ChNPs, TMC has been conjugated with carboxymethyl dextran (CMD) by Nikkhoo et al. (2020) to produce NPs (TMC-CMD NPs) that would serve as carriers for co-delivery of BV6 (an antagonist of Inhibitors of Apoptosis Proteins (IAPs)), small interfering RNA (siRNA) targeting the oncogene Signal Transducer and Activator of Transcription 3 (STAT3), and siRNA targeting NF-κB-Inducing kinase (NIK). This combinatorial approach aimed to block the IAPs that are overexpressed in cancer, mitigate BV6-induced side effects, and suppress STAT3 expression, which has been shown in vitro to induce anticancer responses in breast cancer, colorectal cancer, and melanoma cell lines. NIK inhibition was carried out to reduce the unexpected side effects of BV6, which can cause NIK accumulation, enhancing metastasis of some cancer cells. TMC increased the stability of the NPs in physiological conditions, delaying degradation even after 24 h of exposure to serum. Conjugation with CMD increased the solubility and stability of the NPs, enhanced cell transfection, and prevented aggregation by reducing the occurrence of O-methylation, which takes place when the degree of methylation is high (>65%) and could lead to the reduced solubility of NPs and contribute to the responsiveness of NPs due to the presence of carboxyl and hydroxyl groups [43]. Importantly, by maintaining a moderate degree of methylation (34%), the TMC-CMD-NPs exhibited notable stability in blood serum and demonstrated high transfection efficiency (>70%) in various cancer cell lines in vitro. This combined therapeutic approach exhibited promising anticancer efficacy in cancer cell lines, warranting further in vivo validation studies [45].

Another approach is the conjugation of TMC with alginate (ALG) performed by Rostami et al. (2020). The NPs produced were used for siRNA delivery targeting sphingosine-1-phosphate receptor 1 (S1PR1) and the gene for glycoprotein 130 (GP130), both associated with suppression of the transcription of STAT3 and other signaling molecules associated with cancer progression. Alginate, known for its biodegradability and biocompatibility, was electrostatically bound to TMC to facilitate efficient siRNA incorporation and delivery into breast, colorectal, and melanoma tumor cells. The resulting ALG-TMC NPs exhibited high stability in serum, efficient siRNA loading, and significant transfection in cancer cells (>70% in vitro). Furthermore, targeting S1PR1 and GP130 yielded pronounced anti-tumor effects, underscoring the potential of this strategy. Nonetheless, further investigations in animal cancer models are suggested to evaluate the translational potential of this combined therapy in vivo [46].

Furthermore, Ch’s water solubility can be enhanced by forming salts with both organic and inorganic acids. Several studies have investigated the effects of various Ch salts, such as Ch hydrochloride (CHy), Ch lactate (CL), Ch acetate (CAc), Ch aspartate (CAs), and Ch glutamate (CGl), on Ch/DNA complexes and transfection efficiency, and claimed that they could be employed as safe gene delivery vectors [47,48]. Another Ch salt, carboxymethyl Ch (CMC), which is produced when Ch is carboxymethylated, shows better solubility in physiological and alkaline pH, easier cellular uptake, and enhanced antibacterial and anti-inflammatory activity [49]. Carboxyl groups have a pKa of about 4.5, so they acquire a negative charge at physiological pH due to the deprotonation of carboxyl groups, hence increasing the solubility of CMC NPs [8]. At the same time, CMC is positively charged in the acidic tumor microenvironment because of the protonation of amino groups [50]. CMC can be further modified with other molecules or polymers, such as PEI, to construct gene delivery vectors with advocated efficiency [26,50]. PEI is a synthetic cationic polymer that possesses strong positive charge and has been widely used for nucleic acid delivery. PEI-based NPs displayed advanced transfection efficiency but have been considered less safe than ChNPs. Therefore, conjugating PEI to Ch yields polyplexes with enhanced transfection efficiency while preserving a minimum cytotoxicity level [41].

Other Ch derivatives that have been employed for the improvement of transfection efficiency in cancer cells are PEGylated Ch, glycol Ch (GC), Ch oligosaccharide lactate (COL), N-2-hydroxypropyl trimethyl ammonium chloride chitosan (N-2-HACC), and thiolated chitosan (TC). These derivatives will be discussed in more detail in the following sections, since they either provide different functionalities (e.g., increased cellular uptake) or have been combined with other polymers, targeting ligands or peptides to further improve the transfection efficiency of the final carriers. An overview of the different Ch derivatives included in this review is provided in Table 1.

#### 3.1.2. PEGylation

Another frequently used approach to increase the stability of ChNPs in vivo is the addition of polyethylene glycol (PEG) groups to their surface, i.e., PEGylation [44,51,52,53,54]. PEGylation reduces cytotoxicity, enhances stability in body fluids, and prolongs circulation half-life, making it a viable option for gene delivery applications [55]. Additionally, it provides a reduced surface charge, as it reduces the excessive positive charge remaining on the surface of ChNPs, which, in excess, can interact with red blood cells and cause aggregation or even hemolysis [56]. However, this method presents a challenge known as the “PEG dilemma”, since although it produces significant advantages, it can hinder uptake by cells, as well as the subsequent escape of the internalized complex from the endosomes, thus having a negative impact on transfection efficacy [57]. Moreover, the use of PEG may generate antibodies against PEG that destroy the gene carrier, compromising its therapeutic effectiveness [8]. Furthermore, it was found that the degree of PEGylation on the backbone of Ch (PEG graft density/degree of substitution) had an impact on the cellular uptake and gene transfection efficiencies of the PEG-Ch/siRNA nanocomplexes. Gene knockdown efficiency declined as the degree of substitution increased [41]. In most recent studies, PEGylation of ChNPs is applied in combination with other modifications to develop more efficient carriers. Those include targeting ligands [58,59,60] or factors to enhance cell penetration [56].

### 3.2. Strategies for Increasing Cellular Uptake

ChNPs demonstrate a relatively poor cellular uptake, leading to insufficient transfection efficiency. Hence, various approaches have been employed to enhance cellular uptake of NPs by cancer cells and ultimately transfection efficacy. One such strategy involves the conjugation of NPs with cell-penetrating peptides (CPPs), also referred to as “Trojan Horses”, which are distinguished by their ability to translocate though various biological membranes [61]. Other strategies that can boost the cellular uptake of ChNPs involve the chemical modification of Ch with the insertion of guanidine groups that increase its translocation ability (e.g., guanidinylation of O-carboxymethyl Ch, OCMCS) or the conjugation of molecules with CPP-like effect, such as oligoarginine, to Ch derivatives.

#### 3.2.1. Use of Cell-Penetrating Peptides (CPPs)

The first CPP that was discovered in 1988 is HIV-1 TAT, and since then, it has been widely used in the formulation of ChNPs with augmented cellular penetration for gene delivery [56,62,63]. The TAT peptide has a positively charged region capable of binding to cellular DNA and is rich in hydrophilic arginine, facilitating the transport of various biomolecules, including proteins, peptides, plasmid DNA, and oligonucleotides, into cells [64]. The lysine and cationic residues of arginine amino acids are crucial factors in transporting charged molecular loads across biological membranes [65]. This strategy is usually implemented in PEGylated NPs to combine improved stability with increased cellular uptake.

Conjugation of the TAT peptide to the surface of NPs prepared from Ch grafted poly-(N-3-carbobenzyloxy-lysine) (CPCL) was found to improve not only the cell uptake of the resulting carriers, but also the simultaneous transportation of drug (DOX) and genetic material (plasmid p53) to the nucleus of cancer cells. This resulted in an increase in transfection efficiency and apoptosis of HeLa cells when tested in vitro [66].

Allahyari et al. (2021) applied the technique of employing CPPs to develop PEGylated Ch lactate (CL) NPs conjugated with the TAT peptide. CL NPs have been studied extensively as carriers, showcasing improved biological and physicochemical properties. In a previous study by the same researchers, PEG-CL NPs loaded with siRNA demonstrated effective transfection of cancer cells. However, as mentioned above in the PEG dilemma, while PEGylation can enhance NP stability, it often compromises transfection efficiency. To address this issue, the TAT peptide was attached to the PEG group of NPs to enhance cellular uptake. CL-PEG-TAT NPs (CLP-TAT NPs) were evaluated for siRNA transport targeting tumor-promoting factors (CD73 and IL-6), revealing enhanced uptake of CLP-TAT-siRNA NPs in vitro, exceeding 73% in breast and colorectal cancer cells (4T1 and CT26), a significant increase compared to NPs devoid of TAT addition (23%). Furthermore, both in vitro and in vivo studies in murine models bearing breast and colorectal cancer demonstrated reduced expression of siRNA targets in tumor regions, accompanied by diminished tumor size and increased survival rates, underscoring the effectiveness of transfection enhanced by TAT peptide attachment [56].

#### 3.2.2. Other Molecules with CPP-like Effect

For increasing the cellular uptake of ChNPs, other molecules, either Ch derivatives or peptide ligands attached to Ch derivatives, have also been researched. One such molecule is guanidinylated O-carboxymethyl Ch (GOCMCS) used by Tang et al. (2020) to develop a novel gene delivery system along with poly-β-amino ester (PβAE) for delivering survivin-targeting siRNA in lung cancer cells (A549). Survivin, an overexpressed protein in cancer cells, inhibits apoptosis. OCMCS exhibits superior physicochemical and biological properties compared to Ch, such as solubility in aqueous solutions, while PβAE serves as a biocompatible polymer well-studied for gene delivery [42,67]. Guanidinylation of OCMCS was performed to increase cellular uptake by leveraging the translocation ability of guanidine groups, similarly to those found in the arginine residues of CPPs. A previous study by the same researchers demonstrated that GOCMCS nanocarriers exhibited enhanced transfection efficiency compared to their non-guanidinylated counterparts [68]. PβAE/siRNA/GOCMCS NPs exhibited increased cellular uptake in cancer cells in vitro, compared to NPs lacking GOCMCS, indicating that guanidine’s CPP-like effect enhanced NP uptake. Moreover, the presence of GOMCS mitigated the excessive positive charge of the NPs, resulting in a final zeta potential of +12.2 ± 4.94 mV, thus facilitating their uptake via endocytosis. Cellular uptake is faster for positively charged NPs, in contrast to neutral or negatively charged NPs, due to the electrostatic interactions developed with the negatively charged cell membrane [35]. In vitro studies in cancer cells demonstrated apoptosis induced by efficient siRNA delivery leading to survivin gene silencing [69].

Another molecule that was conjugated to ChNPs in order to increase their cellular uptake was an oligoarginine, specifically nona-arginine. Jeong et al. (2021) prepared ChNPs from glycol Ch (GC), a water-soluble Ch derivative, for the delivery of siRNA targeting cyclophilin B. Nona-arginine was conjugated to GC via a spacer arm of ten glycine units to facilitate the interaction of the oligoarginine with the cell membrane. In this study, nona-arginine’s role was dual; it effectively complexed siRNA, since GC is not positively charged, and it significantly increased the uptake from HeLa cells, as well as the efficiency of gene silencing in vitro [70].

### 3.3. Strategies for Enhancing Cell Targeting

In an effort to augment transfection efficiency, and at the same time minimize the potentially harmful side effects of gene therapy, extensive research efforts have been devoted to achieving the specific delivery of genes [39]. The most distinguished pathways utilized by delivery systems to enter cells and transport their contents to a particular destination are receptor-mediated mechanisms. Thus, the specificity of gene delivery can be enhanced by modifying the structure of ChNPs to incorporate targeting ligands [71]. This involves selecting overexpressed molecular markers on the surface of cancer cells as receptors for the ligands incorporated into ChNPs, thereby directing nanocarriers towards specific targets through receptor recognition. Such targets can be the epidermal growth factor receptor (EGFR), cluster of differentiation 44 (CD44) receptors, the asialoglycoprotein receptor (ASGPR), and folate receptors. This active targeted delivery mechanism enables the precise delivery of anticancer agents and gene therapies [72]. The targeting ligands include proteins, aptamers, carbohydrates, peptides, or other small molecules, and their incorporation results in active targeting, thereby improving delivery efficiency and minimizing systemic side effects [73].

An alternative strategy for targeted gene and drug delivery to cancer cells involves releasing incorporated molecules in response to stimuli. NPs can be designed to respond to specific stimuli, both external and internal, that are characteristic of the tumor microenvironment. This enables controlled release of genes/drugs solely in targeted cancer cells, while sparing healthy cells. An example of an internal stimulus is pH. The pH values of normal tissues and blood are neutral (around 7.4), while pH values in tumor tissues are slightly acidic (6.5–7.2). Moreover, lysosomes are even more acidic, with pH 4.5–5.5. In this regard, pH-responsive NPs can be tailored to exploit the slightly acidic pH of the tumor microenvironment, facilitating targeted release of the encapsulated payload [26]. External stimuli, such as ultrasound, have also been utilized for targeted release of therapeutic agents from ChNPs [27].

#### 3.3.1. Use of Targeting Ligands

Hyaluronic acid (HA) can be used to target cancer cells where Cluster of Differentiation 44 (CD44) receptors are overexpressed, due to HA’s high affinity for these receptors. In a study performed by Liang et al. (2021), ChNPs modified with hyaluronic acid dialdehyde (HAD) were prepared to target bladder cancer cells. The addition of dialdehyde groups facilitated the direct binding to the amino groups of Ch, ensuring homogeneous modification of NPs. The resulting Ch-HAD NPs served as carriers for siRNA targeting the Bcl2 oncogene, aiming to inhibit the oncogene and, consequently, the growth of the tumor. In vitro studies demonstrated enhanced uptake of siRNA-Ch-HAD NPs by cancer cells, as indicated by the significant CD44 protein expression following the receptors’ activation, and superior silencing efficiency compared to a commercial transfection reagent (HiPerFect) and NPs with HA instead of HAD (i.e., siRNA-Ch-HA NPs). Indicative of the targeted gene silencing and successful siRNA delivery of siRNA-Ch-HAD NPs was the efficient inhibition of Bcl2 protein expression. Moreover, the NPs exhibited stability in serum, low cytotoxicity, and effective endosomal escape. In vivo studies performed in tumor-bearing nude mice, showed that siRNA-Ch-HAD NPs effectively targeted tumor cells, resulting in reduced Bcl2 protein levels and inhibited tumor growth, indicating their potential for bladder cancer therapy [74].

Another targeting ligand that can be used in ChNPs to improve cell specificity is lactobionic acid (LA). LA can target cancer cells, such as hepatocellular carcinoma (HCC) cells, expressing the asialoglycoprotein receptor (ASGPR) on their cell membrane, by utilizing β-galactose. Zhang et al. (2020) prepared LA-bearing ChNPs incorporating paclitaxel (PTX) and the plasmid of the single-guided vascular endothelial growth factor receptor 2 (sgVEGFR2)/Cas9 (VC) to achieve synergistic anticancer effects. In vitro studies demonstrated enhanced accumulation of LA-coated ChNPs within ASGPR-overexpressing cells, coupled with increased gene processing efficiency and reduced expression of VEGFR2 and NF-kB p65 protein associated with angiogenesis. In vivo studies in an HCC mouse model revealed more than 70% inhibition of tumor growth and over 33% gene processing efficiency. Notably, the prepared NPs exhibited enhanced stability and low in vivo toxicity, suggesting their potential for clinical translation [75].

An alternative approach is the usage of high-density lipoprotein (HDL) as a ligand in ChNPs to selectively target liver cancer cells that overexpress the scavenger receptor class B type 1 (SR-B1), through which the internalization of HDL particles takes place. These NPs were used to deliver Bcl-2 siRNA, with the aim of inhibiting Bcl-2 protein synthesis and promoting apoptosis of cancer cells. The results indicate that HDL can effectively be used as a targeting ligand, since HDL/Ch/siRNA NPs exhibited increased apoptosis of human liver cancer cells (HepG2) in vitro, compared to ChNPs without HDL, thus indicating high transfection efficiency [76].

Targeting ligands can also be combined to achieve improved targeted delivery to cancer cells. In a study conducted by Khademi et al. (2022), two ligands, HA and the aptamer AS1411, were incorporated on the ChNPs’ surface to target CD44 receptors and nucleolin respectively, to effectively deliver CRISPR/Cas9 to cancer cells. The NPs produced aimed to suppress the expression of FOXM1, an oncogenic transcription factor overexpressed in various cancers. In vitro studies demonstrated superior transfection efficiency in cancer cells (MCF-7, HeLa, SK-MES-1,) compared to non-targeted control cells (HEK293). Moreover, in vivo experiments in tumor-bearing mice revealed significant inhibition of tumor growth and increased survival rates, underscoring the enhanced tumor targeting ability of the nanoconjugates with reduced off-target effects [77].

#### 3.3.2. Stimuli Responsive ChNPs

Since the pH of tumor tissues is lower than that of plasma, ChNPs can target cancer cells by being able to respond to pH changes and release their contents when found in a slightly acidic environment. This can be obtained through the conjugation of Ch with the polymer PEI, which has been extensively studied for its gene carrier properties due to its ability to enhance transfection efficiency in gene therapy. This system, developed by Yan et al. (2020), was formulated to co-administer doxorubicin (DOX) and siRNA to hepatocellular carcinoma cells. DOX was tethered to Ch-PEI NPs via a pH-sensitive hydrazinobenzoic acid linker, resulting in the formation of a micellar prodrug structure. This structure encapsulated hydrophobic DOX within the inner core, with the polymer forming the outer shell. Additionally, the Ch-PEI shell encapsulated siRNA targeting the Bcl-2 gene. For enhanced specificity to cancer cells, glycyrrhetinic acid (GA) was attached to the nanoparticles to target GA receptors, often overexpressed on the surface of hepatocellular carcinoma cells. Consequently, GA-Ch-PEI-HBA-DOX-siRNA nanoparticles were formed. The main results of this study demonstrated effective delivery of both DOX and siRNA to the tumor site in vitro and in vivo, leading to enhanced anticancer activity compared to the administration of the agents separately. The addition of GA significantly increased the uptake of NPs in cancer cells compared to Ch-PEI NPs without GA. The pH-responsive nature of the carrier allowed for the specific release of both therapeutic agents (>90%) in the acidic tumor microenvironment (pH = 5.5), thereby reducing systemic toxicity and increasing therapeutic efficacy. To further investigate the in vivo therapeutic outcomes of the NPs in liver tumors, BALB/c nude mice inoculated with HepG2 tumors served as animal models. The study demonstrated that GA-Ch-PEI-HBA-DOX-siRNA NPs exhibited the highest therapeutic effect compared to free DOX or GA-Ch-PEI-HBA-DOX NPs without siRNA, with tumor inhibition rates of 88%, ~60%, and 79.8%, respectively. Moreover, the polymeric carrier exhibited favorable biocompatibility and stability in serum, indicating its potential as a safe and effective drug–gene co-delivery system for cancer therapy [26].

#### 3.3.3. Biomimetic NPs

A different approach to increasing the active targeting of ChNPs is coating them with macrophage membranes, due to the functional proteins being retained on the membrane. This technique also increases the circulation time of the final carriers. Biomimetic ChNPs were constructed by Li et al. (2022) for the treatment of oral squamous cell carcinoma (OSCC), where ChNPs complexed with miR-451a were coated with exosomes produced by M2 macrophages (MEXO) transfected with miR-144-5p. Thus, the final system was used for the cooperative delivery of miR-144/451a clusters and was found to have successfully protected the encapsulated miRNAs from enzymatic degradation and reduced cell invasion and proliferation and migration of OSCC, due to the inhibitory effects of the encapsulated miRNAs [78].

### 3.4. Strategies for Facilitating Endosomal Escape

A critical step in nucleic acid release from ChNPs into the cytoplasm of target cells is the escape of the delivery system from endosomes through the “proton sponge” mechanism. However, showed that the low buffering capacity of Ch results in a relatively weak endo-lysosomal escape phenomenon, leading to low transfection efficiency. To address this issue, various strategies have been developed to modify ChNPs for more effective endosomal escape. One of these approaches involves the incorporation of poly(propyl acrylic acid) (PPAA), a pH-sensitive polymer presenting strong endosomal membrane-disrupting ability, in ChNPs, thus facilitating gene release into the cytoplasm. In a study conducted by Huang et al. (2020), a series of reconstructed Ch polycations with alkylamines (alkylamine-Ch, AA-Ch) were designed to optimize the properties of Ch, particularly its buffering capacity. The polymers produced were propylamine (PA)-Ch (PA-Ch), (diethylamino) propylamine (DEAPA)-Ch (DEAPA-Ch), and N,N-dimethyl-dipropylenetriamine (DMAPAPA)-Ch (DMAPAPA-Ch), and their buffering capacity under endosomal conditions was evaluated. The buffering capacity of AA-Ch was found to be enhanced compared to Ch, with DMAPAPA-Ch showing the highest buffering capacity (1.6 times higher than Ch), due to the presence of multiple amino groups. DMAPAPA-Ch demonstrated better cellular uptake and endosomal escape, resulting in enhanced transfection efficiency. The researchers examined DMAPAPA-CS in human lung cancer cell lines (A549) and found it effectively delivered the therapeutic plasmid p53 into the cells. Effective expression of the plasmid induced apoptosis in A549 cells, while in vivo studies in mice bearing A549 tumors showed that the DMAPAPA-CS complex with p53 plasmid DNA was able to penetrate the tumor tissue and significantly inhibit its progression [24].

### 3.5. Synergistic Approaches

#### 3.5.1. Combining Enhanced Stability and Targeting

##### PEGylated ChNPs with Targeting Ligands

Ch and its derivatives offer versatile platforms for the construction of NPs with enhanced stability and targeting capabilities. By conjugating other polymers to Ch and functionalizing its chains with various ligands and stimuli-responsive molecules, researchers have devised sophisticated delivery systems for gene delivery to tumor cells.

Afrouz et al. (2022) investigated the coupling of cationic polymers and polylactic acid (PLA)-PEG, with Ch and folic acid (FA) to prepare NPs with enhanced biological and physicochemical properties, for efficient DNA delivery to cancer cells. PLA-PEG NPs have been used as gene carriers, as they exhibit high biocompatibility and circulation time, but low efficiency in gene delivery to cells. PLA-PEG/Ch-FA/DNA NPs were prepared with different ratios of PLA-PEG and Ch-FA (Ch-FA/PLA-PEG2:30, 6:30, 15:30, and 30:30). The study demonstrated that increasing the concentration of FA in the NPs increased zeta potential, DNA transport and release, and transfection efficiency in vitro in breast cancer cells, with DNA expression rates up to 38.22% for the highest Ch-FA ratio (Ch-FA/PLA-PEG 30:30). Cell internalization is further facilitated by the increased zeta potential, which enhances the stability of NPs by fostering electrostatic repulsions among similarly charged particles. This charge-mediated repulsion prevents aggregation, thereby maintaining the NPs’ size. Moreover, since cell membranes carry a negative charge, the positive charge of the NPs promotes interaction with the membranes, facilitating cellular entry. Consequently, this phenomenon plays a pivotal role in enhancing cellular transfection efficiency. The presence of PEG contributed to NPs’ stability by increasing the solubility of Ch, while FA facilitated targeting of cancer cells through binding to the overexpressed folate receptors. Notably, NPs with the highest Ch-FA ratio exhibited the most efficient DNA delivery to cancer cells in vitro [59].

FA was also used in PEGylated ChNPs for targeted siRNA delivery to glioblastoma, one of the most common primary brain tumors, in a study performed by Fukui et al. (2020). In this case, for the preparation of the NPs, Ch oligosaccharide lactate (COL) was used and conjugated with PEG and FA. Ch oligosaccharide is an oligomer of Ch with a lower average molecular weight < 5000 Da, which possesses favorable physical-chemical properties, such as better water solubility and being cationic at neutral pH [79]. Conjugation of Ch oligosaccharide with lactic acid further improves water solubility. FA incorporation led to enhanced targeting of glioblastoma cancer cells due to the overexpression of folate receptor 1 (FOLR1), also evident in several other cancers. The FA-PEG-COL NPs exhibited increased in vitro cellular internalization compared to NPs lacking FA, along with accumulation in tumor area in vivo. These NPs successfully delivered siRNA targeting the CD146 gene, leading to reduced CD146 expression, effective cell lysis, and significant suppression of intracranial tumor growth in a mouse glioblastoma model. Moreover, the NPs managed to penetrate the blood–brain barrier via the EPR phenomenon. However, further research is warranted to optimize strategies for gene delivery to brain tumors [60].

In a study performed by Salimifard et al. (2020), PEGylated NPs from CL with incorporated HA to improve targeting via binding to CD44 receptors were prepared for the co-delivery of BV6 and siRNA targeting interleukin-6, leveraging previous findings on the enhanced physicochemical and biological properties of ChNPs’ post-reaction with lactic acid and PEGylation. Results demonstrated high efficiency in cancer cell transfection, achieving a rate of 75% with NPs conjugated with HA, owing to targeted binding to CD44 receptors. Conversely, in a cell culture medium pre-treated with HA, receptor occupation limited NP transfection to only 18%. Additionally, significant anticancer effects were observed both in vitro and in vivo in mice bearing 4T1 (breast) and CT26 (colon) tumors, where suppression of the tumors’ growth was obtained [58].

##### Ch Derivatives with Targeting Ligands

Apart from PEGylated ChNPs, targeting ligands can be combined with NPs of increased stability produced by chemically modified Ch polymers, such as TMC. Masjedi et al. (2020) utilized TMC NPs conjugated with HA for the co-delivery of siRNAs targeting interleukin 6 (IL6) and the oncogenic transcription factor STAT3, both crucial in cancer progression. The final NPs were found to be stable under physiological conditions (i.e., serum and simulated gastric environment with pH 1.6), were not cytotoxic, and had a high encapsulation efficiency. In terms of anticancer activity studied in vitro, the NPs exhibited increased cellular uptake and controlled release of the encapsulated siRNA, as was evident through the high percentage of cell lysis (>70%) in cancer cells overexpressing CD44 which are targeted by HA, and inhibition of IL-6 and STAT3 expression (90%), with the latter resulting in halting the progression of cancer cells, metastasis and angiogenesis. These results demonstrate the importance of CD44 targeting by HA in enhancing transfection efficiency [80].

Additionally, TMC NPs were employed for the delivery of human SET1 antisense oligonucleotide, known for its anticancer activity. For achieving targeted delivery to cancer cells, a series of anionic molecules were complexed to TMC to produce polyelectrolyte NPs, specifically HA, alginate, or dextran sulfate, to find the combination with the best physicochemical and anticancer properties. Among these, HA-bearing NPs demonstrated the most promising results. Subsequent in vitro and in vivo studies confirmed NP accumulation in cancer cells, attributed to the CD44-targeting property of HA, thus enhancing NP uptake into cancer cells, low cytotoxicity, minimal accumulation in other organs, and up to 44% reduction in SET1 expression post-cancer cell lysis [81].

Another Ch derivative with improved solubility that produces stable ChNPs and has been combined with targeting ligands for enhanced cancer cell targeting is carboxymethyl Ch (CMC). In a study performed by Chen et al. (2020), CMC was used to prepare NPs conjugated with N-2-hydroxypropyl trimethyl ammonium chloride Ch (N-2-HAAC), a cationic Ch derivative with improved solubility, and FA for increased targeting. These NPs effectively delivered STAT3-targeting siRNA in lung cancer cells, exhibiting increased cellular uptake (75%) attributed to folic acid-mediated receptor binding and endocytosis. Notably, cancer cell lysis obtained with these NPs resulted in up to 45% reduction in tumor volume in vivo, accompanied by significant suppression of STAT3 protein expression both in vitro and in vivo. This study underscores the potential of enhancing stability and targeting of ChNPs to improve the efficacy of cancer cell lysis, for targeted delivery of STAT3-targeting siRNA to lung cancer cells [82].

##### Ch Derivatives with Stimuli-Responsiveness Ability and Targeting Ligands

The synergistic approach of combining TMC with a targeting ligand (e.g., FA) can be further enhanced by an additional structural modification, rendering the system responsive to pH change and able to release its content upon exposure to the acidic pH of the tumor microenvironment. This strategy was employed by Zhang et al. (2021), who fabricated NPs of TMC conjugated with carboxymethyl-β-cyclodextrin (CMCD) and FA (FCT NPs). CMCD provided a hydrophilic shell and a hydrophobic cavity for incorporating various hydrophobic molecules, thereby improving their physicochemical and biological properties, such as increased solubility and stability, reduced toxicity, improved bioavailability, and controlled release. The anticancer drug DOX was incorporated into the hydrophobic cavity of CMCD, and siRNA was incorporated into TMC. The transfection of human lung adenocarcinoma cell lines was then studied. Based on the results of in vitro studies, the toxicity induced in cancer cells after transfection with the NPs under study increased, attributed to targeting due to FA, indicating effective transfection of cancer cells. Additionally, the NPs exhibited satisfactory stability in serum, while a significant increase in the release rate of DOX and siRNA was observed at acidic pH. Specifically, the total release rates of DOX after 192 h in buffer solutions with pH 7.38 and pH 5.29 were ~39% and 80%, respectively. Similarly, the release of siRNA at pH 5.29 was 71%, while at pH 7.38, it was only ~23%. This phenomenon is related to the swelling of FCT NPs induced by the protonation of the TMC skeleton in acidic medium, indicating targeted drug release with pH responsiveness, leading to drug accumulation in tumor tissues and reduction of side effects on normal tissues [72].

pH-triggered targeted release by protonation in the acidic tumor environment can also be obtained with the utilization of 2-(diisopropylamino) ethyl methacrylate (DPA) in combination with TMC, for increasing the stability of NPs, and FA, for increasing cell targeting. This system was constructed by Li et al. (2022) for the co-delivery of DOX and CRISPR/Cas9 plasmid expressing survivin (Survivin CRISPR/Cas9-expressing plasmid -sgSurvivin pDNA) or plasmid expressing shRNA survivin (Survivin shRNA-expressing plasmid, iSur pDNA) to reduce survivin protein expression, involved in inhibiting cancer cell apoptosis. The results demonstrated high efficiency in delivering the payload to the nucleus of cancer cells, reaching approximately 80%. In vivo, after intravenous administration to mice bearing breast tumors, survivin expression in tumor tissue was significantly lower than in the control group, which received only normal saline (approximately 37% versus 68.4%). Furthermore, significant tumor inhibition was observed from the administration of nanoparticles with DOX and FA, compared to the group receiving treatment with free DOX (70.6% versus 50.9%). In conclusion, FTD/DOX/sgSurvivin pDNA and FTD/DOX/iSur pDNA NPs exhibited anticancer effects to a similar extent, good structural stability, pH-responsive drug release, and enhanced cancer cell targeting [83].

An alternative strategy in targeted drug delivery via stimulus–responsive release is the development of polymeric systems that respond to external stimuli and, more specifically, to ultrasound. Such a system was developed by Yan et al. (2020) by combining CMC and Ch hydrochloride (CHC) for the targeted delivery of siRNA in colorectal cancer cells. CMC and CHC are water-soluble derivatives of Ch, and the NPs constructed from them exhibit enhanced stability. The siRNA they delivered targeted the β-catenin gene, which is overexpressed in the majority of colorectal cancer cells and has been associated with their proliferation. Based on the results from in vitro studies, a high release of siRNA from the nanoparticles was observed (56.04%) upon ultrasound exposure for 8 min after adjustment to pH 5.5, simulating the microenvironment of the tumor, as well as further sustained release up to 70.07% after 120 min. The release of siRNA at pH 1.2–2.2 was very low (11%), indicating that the NPs were stable and could effectively delay the release of siRNA in extremely acidic environments simulating gastric pH. Overall, the effective, targeted, and controlled release of siRNA from the NPs was attributed to response to two different stimuli, the internal stimulus of pH (pH 5.5 simulating the environment of the colon tumor), and the external stimulus of ultrasound. Additionally, the levels of β-catenin after transfection with these NPs were reduced to 40% compared to those in control samples, indicating a good internalization of siRNA into cancer cells. Further in vivo studies are required for proving the enhanced efficiency of these ChNPs in the targeted oral delivery of siRNA to colorectal cancer cells through ultrasound processing [27].

#### 3.5.2. Combining Targeting and Enhanced Endosomal Escape

An effective strategy for improving cancer treatment in multidrug-resistant (MDR) cancers, such as breast cancer, involves utilizing a pH-responsive targeted Ch polymeric system to co-deliver an anticancer drug (DOX) along with MDR1 gene-silenced siRNA (siMDR1). In a study performed by Peng et al. (2022), a cell-targeting pH-responsive system was prepared comprising of a CMC core carrying DOX conjugated via a disulfide bond, and siMDR1 encapsulated in oligoethylenimine (OEI), associated with the polymeric core via electrostatic interactions. Two ligands were used for targeting purposes, i.e., GALA peptide-conjugated hyaluronic acid (GHA) and AS1411 aptamer-conjugated hyaluronic acid (AHA). GHA not only aids in targeting but also enhances endosomal escape due to the change in GALA conformation from random coil to a-helix under acidic conditions, leading to endosome destabilization. Furthermore, the release of siMDR1 and DOX occurs sequentially, with siMDR1 being released after endocytosis due to CMC protonation and DOX being released subsequent to endosomal escape in the cytoplasm, facilitated by glutathione-mediated cleavage of the respective disulfide bonds. Effective gene silencing (86%) was achieved in vitro in MCF-7/ADR cells followed by significant cell apoptosis (56%), while a significant reduction of tumor size could also be observed in vivo in MCF-7/ADR tumor-bearing mice [84].

A different strategy is usage of a Ch derivative modified with multiple histidine groups for enhancing endosomal escape in combination with monoclonal antibodies for cancer cell targeting. Histidines contain imidazole groups that can be protonated at the acidic pH of endosomes, thus creating a proton sponge effect that is important for endosomal escape. Specifically, Zhang et al. (2022) prepared ChNPs from CMC altered by histidine cholesteryl ester, with the addition of cholesterol, to promote NP self-assembly and enhance cellular uptake, and epidermal growth factor receptor (EGFR) antibodies for targeting esophageal squamous cancer cells. The developed system encapsulated three different agents: adriamycin, as a cancer chemotherapeutic, as well as MVP-siRNA and BCL2-siRNA to inhibit the major vault protein (MVP) and the B-cell lymphoma-2 (BCL2) gene which are responsible for the multidrug resistance exhibited by the specific cells. From both in vitro and in vivo studies performed in the adriamycin-resistant cell line (510 K cells) and in mice bearing the respective tumor, it was demonstrated that the NPs had improved cellular uptake in cancer cells and release of the encapsulated active agents in the cytosol, and significantly improved cell apoptosis (66.2%), indicating the successful transfection efficiency of both encapsulated siRNA andriamycin, and successful suppression of tumor growth in vivo [85].

#### 3.5.3. Combining Enhanced Stability, Targeting, and Extensive Cellular Uptake

In efforts to engineer more stable, cell-specific, and efficient ChNPs for gene delivery to cancer cells, Salehi Khesht et al. (2021) conjugated HA to CL NPs, for specific targeting of cancer cells through CD44 receptors, and the TAT peptide, for enhanced entry into cancer cells. The nanocarriers were investigated for efficient delivery of DOX and CD73 siRNA to cancer cells (4T1, CT26), both in vitro and in vivo, using BALB/c mice models. CD73 is highly expressed in tumor microenvironments, and is essential for primary cancer cell functions like proliferation and angiogenesis, making it a vital anticancer target. In vitro studies of the investigated NPs demonstrated high cancer cell uptake, reaching up to 78%, whereas corresponding NPs without HA and TAT exhibited only 35% uptake. Additionally, effective silencing of CD73, up to 90%, was observed in vitro, accompanied by remarkable anticancer effects from DOX/CD73 siRNA co-delivery. In vivo studies demonstrated NP accumulation in the tumor microenvironment, significant reduction in tumor growth, improved survival in tumor-bearing mice, and induction of anticancer immune responses [64].

In the same context, Bastaki et al. (2021) used thiolated Ch (TC) in combination with TMC, HA, and TAT to construct siRNA-carrying NPs for targeted cancer cell delivery. Modification of Ch with the thiol group in TC significantly enhanced the mucoadhesive properties of Ch and its structural stability, exhibiting favorable drug entrapment ability, high cell penetration rates, and prolonged release properties. The transfer of siRNA was examined in vitro and in vivo using murine cancer models for breast cancer (4T1) and melanoma (B16-F10). In vitro studies demonstrated significant entry of most NPs into cancer cells through the interaction of HA in NPs with CD44 in cancer cells, with the presence of the TAT peptide facilitating cellular uptake. TAT-HA-conjugated NPs exhibited three times higher permeability compared to NPs without TAT-HA, resulting in lysing of 75% of target cells. Furthermore, significant reduction in mRNA and protein levels of PD-L1 and STAT3 molecules was observed in B16-F10 and 4T1 cancer cells. Finally, impressive anticancer results were observed in vitro, with limited tumor growth observed in vivo [86].

## 4. Discussion

Ch is one of the most widely studied biopolymers used as non-viral nucleic acid carriers in gene therapy. As a naturally derived polymer, it presents significant advantages such as biocompatibility, biodegradability, and low toxicity. Its unique properties make it an attractive candidate for delivering therapeutic nucleic acids such as siRNA, DNA, miRNA, CRISPR-Cas9, and shRNA to target cells. Ch’s ability to electrostatically interact with negatively charged nucleic acids allows for the formation of stable complexes, protecting the genetic cargo from degradation by nucleases. This characteristic is crucial for efficient gene delivery, as it ensures the integrity of the payload until it reaches the target cells. Moreover, Ch’s cationic nature facilitates cellular uptake through interactions with negatively charged cell membranes, leading to intracellular delivery of therapeutic nucleic acids.

The success of gene therapy largely depends on the gene delivery system, which must meet the following requirements: cell-targeting ability, low cytotoxicity, high gene delivery efficiency, protection of the gene payload from serum nucleases, sustained gene release, endosomal escape ability, and high transfection efficiency. Gene carriers based on Ch exhibit high therapeutic potential, as they meet most of these required characteristics. However, Ch is positively charged only at low pH and has a relatively low charge density, which negatively affects the physical stability of the Ch-gene complexes in blood pH and the efficiency of Ch to induce gene escape from endosomes after cellular uptake of the Ch-gene complex. To address these limitations, which result in low transfection efficiency [20], numerous strategies for modifying ChNPs have been explored to develop carriers with overall improved transfection efficiency, as summarized in Table 2, with four main strategies and several combinations thereof being identified.

In these reviewed studies, ChNPs have been employed to deliver a wide range of genetic materials, each with distinct therapeutic purposes. The most common payload incorporated into these carriers for cancer gene therapy is small interfering RNA (siRNA), which targets specific mRNA sequences of target cells, causing their degradation or inhibition and ultimately suppressing the synthesis of the target protein [20]. Along with microRNAs and short-hairpin RNAs (shRNA), they play a crucial role in RNA interference (RNAi). siRNA-based gene therapy has garnered more attention due to the simplicity of siRNA production and characterization. siRNAs are smaller in size, making them easier to chemically modify for enhanced nuclease stability, and they can be synthesized in large quantities. Additionally, siRNA-based therapeutics are extensively researched for various diseases, with five siRNA-based drugs already approved by the FDA for conditions such as hereditary transthyretin amyloidosis (hATTR), acute hepatic porphyria (AHP), primary hyperoxaluria type 1 (PH1), primary hypercholesterolemia, and amyloid transthyretin-mediated (ATTR) amyloidosis [87]. Regarding tumor gene silencing, siRNAs have been studied in recent years to treat breast, colorectal, cervical, and bladder cancer, melanoma, lung adenocarcinoma, hepatocellular carcinoma, and glioblastoma. Moreover, many studies aim to co-administer siRNA with anticancer agents such as paclitaxel, doxorubicin, and BV6, and their synergistic action has been demonstrated. Apart from siRNA, miRNAs, non-coding RNAs exerting their influence on target genes at the post-transcriptional level, have also been studied as potential therapeutic agents for cancer treatment, with changes in miRNA levels being found in various tumor tissues [78].

Another cancer therapeutic strategy is the delivery of plasmid DNA, such as p53 plasmid, with ChNPs. p53 plasmid delivery aims at restoring the functionality of this tumor suppressor gene and inducing cancer cell death. Basically, after p53 plasmid is taken up by cancer cells, it is transcribed into mRNA and translated to p53 protein, which exhibits tumor suppressing activity. Mutations in the TP53 gene, which impairs the tumor suppressor functions of the p53 protein, are the most prevalent single gene alterations in human cancers and are considered driver events in numerous tumor types. In this therapeutic approach belongs the first gene therapy product approved for clinical use, Gendicine, which is a recombinant human p53 adenovirus for treating head and neck squamous cell carcinoma [88]. Moreover, antisense oligonucleotides can be delivered with ChNPs to suppress the expression of target oncogenes. For cancer gene therapy, hSET1 is a significant target, since it is overexpressed at both the mRNA and protein levels in malignant cells, which are characterized by high mitotic rate. hSET1 is an essential component of the histone methyltransferase complex, a crucial enzyme in gene expression regulation, that can be downregulated by human SET1 (hSET1) antisense, a 17mer DNA-base oligonucleotide. Targeting this enzyme could disrupt the abnormal gene expression patterns driving cancer progression [46].

A different therapeutic approach that has been utilized in recent studies for cancer gene therapy is CRISPR/Cas9. Compared to RNAi, CRISPR/Cas9 technology employs a single guide RNA (sgRNA) to direct the Cas9 enzyme to a specific DNA locus, where it cleaves the DNA to knockout the target gene. This method achieves more precise gene regulation by inhibiting protein expression slowly and irreversibly, and ChNPs can be an effective delivery system for CRISPR/Cas9 components (either plasmid-encoding Cas9, Cas9 mRNA or Cas9 protein along with sgRNA) due to their ability to protect, effectively carry larger payloads, and target specific cells [83]. Overall, ChNPs can effectively deliver a broad range of genetic materials, showcasing their versatility and potential in cancer treatment. Understanding the specific requirements and challenges associated with each type of genetic material is crucial for optimizing their design and application.

Strategies that aim at increasing the stability of Ch primarily focus on utilization of Ch derivatives that exhibit enhanced solubility in aqueous solutions (especially at physiological pH). Various derivatives can be used, such as Ch lactate, carboxymethyl Ch, N-2-hydroxypropyltrimethyl ammonium chloride Ch, and hydrochloric acid Ch, with TMC being the derivative that was most commonly used. As a new approach, TMC can be combined with other polymers, such as carboxymethyl dextran or alginate [46], to further improve the physicochemical properties of ChNPs and ultimately the transfection efficiency. Indeed, conjugation of Ch with these polymers led to over 70% transfection efficiency in cancer cells in vitro, along with remarkable anticancer results.

Another method applied in several studies to improve the stability of ChNPs was PEGylation, which, however, is known for negatively affecting transfection efficiency. Hence, to optimize transfection, further modification of the PEGylated ChNPs is required. In most studies, PEGylated NPs composed of Ch derivatives were combined with additional strategies to increase cancer cell targeting or enhance cellular uptake, or both.

Targeting of cancer cells relies on the fact that, after the introduction of nucleic acid carriers into the body, it is crucial for them to enter target cells as efficiently as possible, since the target sites for gene therapy are located inside the cells (DNA is expressed in the nucleus, while siRNA targets the cytoplasm). Moreover, targeting is important not only to increase efficiency but also to avoid unwanted actions on healthy tissues. Increased targeting can be achieved by attaching specialized ligands to the surface of ChNPs that will bind to protein receptors that are overexpressed on the surfaces of cancer cells. Thus, internalization of ChNPs to target cells is not only performed via ATP due to electrostatic interactions between the positively charged Ch and the negatively charged cell membrane, but also through endocytosis induced by ligand–receptor interaction. The most commonly used ligand is hyaluronic acid, which targets CD44 receptors overexpressed in cancer cells, while another widely used ligand is folic acid, targeting folate receptors overexpressed in cancer cells. Other ligands include lactobionic acid bearing β-galactose, targeting ASGPR receptors of HCC cancer cells, AS1411 aptamer targeting nucleolin overexpressed in the nucleus of cancer cells, glycyrrhetinic acid targeting glycyrrhetinic acid receptors often overexpressed on the surface of liver cancer cells, and HDL targeting cells overexpressing SR-B1. Another strategy applied in several studies to enhance targeting and controlled release of genes to cancer cells was the utilization of polymers that introduce responsiveness of the ChNPs to internal and external stimuli, i.e., releasing their contents after exposure to certain stimuli, such as pH and externally applied ultrasound. As a result, ChNPs release their genetic payload only in the acidic microenvironment of cancer cells, thus enhancing the selectivity and efficiency of gene transfer. The polymers that have been used to achieve this in combination with a Ch derivative, such as TMC or CMC, were carboxymethyl-β-cyclodextrin (CMCD) [72], 2-(diisopropylamino) ethyl methacrylate (DPA) [83], and Ch hydrochloride (CHC) [27]. The majority of studies that produced stimuli-responsive ChNPs employed a combination of this targeting strategy with some stability enhancement technique (e.g., the usage of Ch derivatives or PEGylation) and the usage of targeting ligands such as HA or FA to further improve targeting of cancer cells and ultimately transfection. The combination of stability and targeting led to highly effective cancer cell transfection rates of up to 80% in vivo. A different strategy to targeting cancer cells involves the coating of ChNPs with macrophage membranes to produce biomimetic NPs [78].

To achieve improved cellular uptake, researchers most commonly employed cell-penetrating peptides, such as the TAT peptide [56,64,86]. Alternatively, guanidinylated O-carboxymethyl Ch can be used for promoting cellular uptake of NPs, due to the guanidinyl groups which contribute to the transmembrane transport of macromolecules in a similar way to CPPs, which also contain guanidinyl groups in their arginine residues [69]. Furthermore, nona-arginine can exhibit CPP-like behavior, leading to enhanced cellular uptake [70]. When CPPs were combined with targeting ligands (HA) in ChNPs with CL, transfection efficiency was improved significantly, reaching 78% in vitro [64].

The final strategy evolves around improving the endosomal escape of ChNPs following internalization to cancer cells. To achieve this, the buffering capacity of Ch was enhanced by linking it with alkylamines. The most effective modification was the conjugation of Ch with N,N-dimethyl-dipropylenetriamine, which resulted in a buffering capacity 1.6 times greater than that of unmodified Ch. Corresponding NPs achieved effective cell lysing, delivery of the therapeutic plasmid p53, and oncosuppressive effects in vitro (A549 cells) and in vivo (A549 tumor-bearing mice), indicating the importance of regulating and optimizing the inherent property of Ch for intracellular escape [24]. This effect was also produced by chemically modifying Ch with histidine groups that are protonated in acidic pH [85], or with conjugating GALA at the surface of ChNPs that change conformation in acidic pH, resulting in endosome destabilization, with both approaches leading to enhanced transfection efficiency both in vitro and in vivo [84].

The reviewed studies underscore the significant progress made in developing ChNPs for cancer gene therapy. However, several challenges remain, including optimizing stability, targeting specificity, cellular uptake, and endosomal escape. Future research directions may involve further refinement of existing strategies, as well as the exploration of novel approaches such as combinatorial strategies for enhanced therapeutic outcomes. Additionally, the translation of preclinical findings into clinical applications will require rigorous evaluation of safety and efficacy in realistic animal models of cancer and successful resolution of scalability issues to meet the demands of cancer patients in a clinical setting. Overall, ChNPs hold high potential for revolutionizing cancer treatment paradigms and improving patient outcomes.

## 5. Conclusions

Despite significant progress in cancer therapy in recent decades, cancer-related mortality remains one of the leading causes of death worldwide. Ch is a natural polymer that has been extensively researched, making it one of the most important polymers that can be used as nanocarriers in gene therapy for cancer. The remarkable anticancer efficacy of optimized nanoparticles developed after judicious modifications of Ch nanoparticles has been demonstrated both in vitro and in vivo, indicating a wide range of clinical applications, such as the delivery of therapeutic nucleic acids, chemotherapeutic drugs, and other substances to various types of cancer cells. Additionally, Ch itself has exhibited inhibitory effects on cancer cells through multiple signaling pathways. However, clinical translation of gene carriers based on Ch for cancer treatment still faces some obstacles. Firstly, regarding recent studies on Ch nanocarriers for cancer gene therapy, experiments in vivo have been conducted in fewer than half of the studies. Conducting in vivo studies is important because, many times, the very promising in vitro results of gene therapy are not validated in vivo, mainly due to genetic variations in living organisms that are not easy to predict and directly affect the action of gene therapeutics. Also, since Ch is not a synthetic material, there is significant variability among individual batches, making it difficult to fully control the characteristics of each batch (e.g., degree of acetylation, molecular weight), which play a significant role in nanoparticle construction. Finally, it is not clear whether the mechanism of Ch nanoparticle gene cargo delivery to cancer cells will function in cancer patients as it does in the rodent tumor models studied [28]. Therefore, extensive in vivo studies in realistic animal models of cancer as well as clinical studies in humans are required, along with more approaches for modifying Ch, to fully exploit its unique nature [20].

## Figures and Tables

**Table 1:** Overview of the improvements obtained with the use of Ch derivatives.

Chitosan Derivatives	Properties/Improvements
Trimethyl Chitosan (TMC)	Soluble across a wide pH range, higher positive charge that enhances cellular uptake Increased stability under physiological conditions, high transfection efficiency
Ch lactate (CL)	Improved solubility and stability, enhanced transfection efficiency
Ch hydrochloride (CHC)	Improved solubility and stability, stimuli-responsive release (pH and ultrasound)
PEGylated Ch	Pros: Improved stability and solubility, prolonged circulation half-life, reduced surface charge Cons: PEGylation can hinder cellular uptake and endosomal escape
Carboxymethyl Ch (CMC)	Improved solubility in physiological and alkaline pH, negatively charged at physiological pH, positively charged in the acidic tumor microenvironment, increased cellular uptake
Guanidinylated O-carboxymethyl Ch (GOCMCS)	Improved solubility, increased cellular uptake, enhanced transfection efficiency
Glycol Ch (GC)	Improved solubility and stability
Ch oligosaccharide lactate (COL)	Improved water solubility, cationic at neutral pH
N-2-hydroxypropyl trimethyl ammonium chloride Ch (N-2-HACC)	Improved solubility, enhanced stability
Thiolated Ch (TC)	Improved stability, enhanced mucoadhesive properties, favorable drug entrapment, high cellular uptake, prolonged release properties

**Table 2:** Summary of strategies adopted to increase the transfection efficiency of cancer cells by ChNPs.

Strategy	Chitosan/ Chitosan Derivative	Other Polymer/Molecule	Targeting Ligand	Cellular Uptake Enhancing Factor	Load	Cancer	Study Type		Ref.
							In Vitro	In Vivo	
Increased stability	TMC	CMD	-	-	siRNA, BV6	breast, colorectal, melanoma	X		[45]
	TMC	ALG	-	-	siRNA	breast, colorectal, melanoma	X		[46]
Increased cellular uptake	CL	PEG	-	TAT peptide	siRNA	breast and colorectal	X	X	[56]
	GOCMCS	PβAE	-	-	siRNA	lung adenocarcinoma	X		[69]
	GC	-	-	nona-arginine	siRNA	cervical	X		[70]
Improved cell targeting	Ch	-	HAD	-	siRNA	bladder	X	X	[74]
	Ch	-	LA	-	PTX, CRISPR/Cas9	hepatocellular carcinoma	X	X	[75]
	Ch	-	AS1411- HA	-	CRISPR/Cas9	Breast, cervical, kidney	X		[77]
	Ch	-	HDL	-	Bcl-2 siRNA	liver	X		[76]
	Ch	PEI-HBA (pH sensitive)	GA	-	DOX, siRNA	hepatocellular carcinoma	X	X	[26]
	Ch	MEXO	-	-	miRNA	oral squamous carcinoma	X		[78]
Facilitating endosomal escape	Ch	alkylamines (PA-CS, DEAPA-CS, DMAPAPA-CS)	-	-	p53 plasmid	lung	X	X	[24]
Increased stability and improved targeting	Ch	PLA-PEG	FA	-	DNA	breast	X		[59]
	CL	PEG	HA	-	siRNA, BV6	breast, colon	X	X	[58]
	COL	PEG	FA	-	siRNA	glioblastoma	X		[60]
	TMC	-	HA	-	hSET1 antisense	breast	X	X	[81]
	TMC	-	HA	-	siRNA	Breast, colorectal, melanoma	X		[80]
	CMC/N-2-HACC	-	FA	-	siRNA	lung	X	X	[82]
	TMC	CMCD	FA	-	DOX, siRNA	lung adenocarcinoma	X		[72]
	TMC	DPA (pH sensitive)	FA	-	DOX, CRISPR/Cas9 pDNA or shRNA	Breast	X	X	[83]
	CHC/CMC (pH sensitive + ultrasound)	-		-	pDNA siRNA	colorectal	X		[27]
Targeting & enhanced endosomal escape	CMC	OEI	GHA/AHA	-	DOX/siRNA	breast (MDR)	X	X	[84]
	CMC	histidine	EGFR	-	Adriamycin/siRNA	esophageal squamous	X	X	[85]
Increased stability, improved targeting & enhanced cellular uptake	CL	-	HA	TAT peptide	DOX, siRNA	colorectal, breast	X	X	[64]
	TMC/TC	-	HA	TAT peptide	siRNA	breast, melanoma	X	X	[86]

TMC: N,N,N-trimethyl chitosan, CMD: carboxymethyl dextran, ALG: alginate, CL: Ch lactate, GOCMCS: guanidinylated O-carboxymethyl chitosan, PβAE: poly-β-amino ester, GC: glycol chitosan, HAD: hyaluronic acid dialdehyde, LA: lactobionic acid, HDL: high-density lipoprotein, HBA: hydrazinobenzoic acid (linker), GA: glycyrrhetinic acid, MEXO: exosomes produced by M2 macrophages, PA: propylamine, DEAPA: (diethylamino) propylamine, DMAPAPA: N,N-dimethyl-dipropylenetriamine, PLA: polylactic acid, FA: folic acid, COL: chitosan oligosaccharide lactate, N-2-HAAC: N-2-hydroxypropyl trimethyl ammonium chloride chitosan, CMCD: carboxymethyl-β-cyclodextrin, DPA: 2-(diisopropylamino) ethyl methacrylate, CHC: chitosan hydrochloride, OEI: oligoethylenimine, GHA: GALA peptide-conjugated hyaluronic acid, AHA: aptamer-conjugated hyaluronic acid, EGFR: epidermal growth factor receptor, TC: thiolated chitosan.
